# ParA’s Impact beyond Chromosome Segregation in Caulobacter crescentus

**DOI:** 10.1128/jb.00296-22

**Published:** 2023-01-24

**Authors:** Inoka P. Menikpurage, Stephanie G. Puentes-Rodriguez, Rawan A. Elaksher, Paola E. Mera

**Affiliations:** a Department of Microbiology, University of Illinois at Urbana-Champaign, Urbana, Illinois, USA; b Department of Chemistry and Biochemistry, New Mexico State University, Las Cruces, New Mexico, USA; University of California San Francisco

**Keywords:** *Caulobacter*, DnaA, ParA, cell cycle, cell length, chromosome replication, chromosome segregation

## Abstract

Maintaining proper chromosome inheritance after the completion of each cell cycle is paramount for bacterial survival. Mechanistic details remain incomplete for how bacteria manage to retain complete chromosomes after each cell cycle. In this study, we examined the potential roles of the partitioning protein ParA on chromosomal maintenance that go beyond triggering the onset of chromosome segregation in Caulobacter crescentus. Our data revealed that increasing the levels of ParA result in cells with multiple origins of replication in a DnaA-ATP-dependent manner. This *ori* supernumerary is retained even when expressing variants of ParA that are deficient in promoting chromosome segregation. Our data suggest that in Caulobacter ParA’s impact on replication initiation is likely indirect, possibly through the effect of other cell cycle events. Overall, our data provide new insights into the highly interconnected network that drives the forward progression of the bacterial cell cycle.

**IMPORTANCE** The successful generation of a daughter cell containing a complete copy of the chromosome requires the exquisite coordination of major cell cycle events. Any mistake in this coordination can be lethal, making these processes ideal targets for novel antibiotics. In this study, we focused on the coordination between the onset of chromosome replication, and the partitioning protein ParA. We demonstrate that altering the cellular levels of ParA causes cells to accumulate multiple origins of replication in Caulobacter crescentus. Our work provides important insights into the complex regulation involved in the coordination of the bacterial cell cycle.

## INTRODUCTION

The survival of bacteria relies on their ability to preserve the integrity of their genome. Maintaining intact copies of the chromosome after each cell division requires temporal and spatial coordination of different cell cycle events. In bacteria, this coordination must be accomplished while the chromosome is being replicated and segregated concurrently (1). Any mistake in temporally coordinating chromosome replication, segregation, and cytokinesis can result in cell death. Although details of each of these processes have been elegantly revealed, mechanistic details of how these events are coordinated with each other, temporally and spatially, remain limited. In this study, we focused on the coordination between the onset of chromosome replication and the onset of chromosome segregation.

The onset of chromosome replication, a major regulatory step in the cell cycle, is catalyzed by the conserved AAA+ ATPase replication initiator DnaA. DnaA-ATP oligomerizes at the origin of replication (*ori*) and opens the double stranded DNA helix, allowing for the replication machinery to assemble and replicate the chromosome bidirectionally (2, 3). The ability of DnaA to initiate chromosome replication is regulated at a multitude of levels that include autoregulation, deactivation, sequestration, titration, and proteolysis (4–6). In addition to catalyzing this essential step for DNA synthesis, DnaA and/or replication initiation have been shown to influence other key cellular events, including chromosome segregation, cell cycle progression, cytokinesis, cell size, and gene expression (7). These various connections expose the complexity of the highly interconnected network that links one of the first committed events in this developmental process, the onset of chromosome replication, with the rest of the cell cycle. However, mechanistic details at the molecular level of how the initiation of chromosome replication links to various cell cycle events have remained limited.

The partitioning system ParABS is involved in the initial steps for chromosome segregation. The ParABS system was first discovered and characterized in the segregation and maintenance of low copy plasmids (8–10). Most bacterial species (>70%) encode the *parABS* system (11). ParABS is composed of 3 parts: a centromere-like chromosomal locus *parS* and 2 DNA-binding proteins, ParA (ATPase) and ParB (CTPase). ParA bound to ATP forms dimers that bind DNA nonspecifically, and are released from DNA through the interaction with ParB (12, 13). Different lines of evidence have demonstrated connections between the onset of replication and segregation (1). For instance, the centromere-like locus *parS* is commonly found near the chromosomal origin of replication, suggesting a potential proximity link between the onset of replication and the onset of chromosome segregation (9, 11, 14). In Bacillus subtilis and Vibrio cholerae, the partitioning ParA protein has been shown to regulate the onset of chromosome replication (15, 16). In B. subtilis, a variant of Soj (ParA) that remains as a monomer was shown to directly interact with DnaA, and inhibit DnaA’s ability to oligomerize at *ori*, thus preventing the onset of chromosome replication (17). Furthermore, the stability of DnaA bound at *ori* increases in B. subtilis with *soj* (*parA*) knocked out, resulting in over-initiation of replication (18).

**FIG 1:**
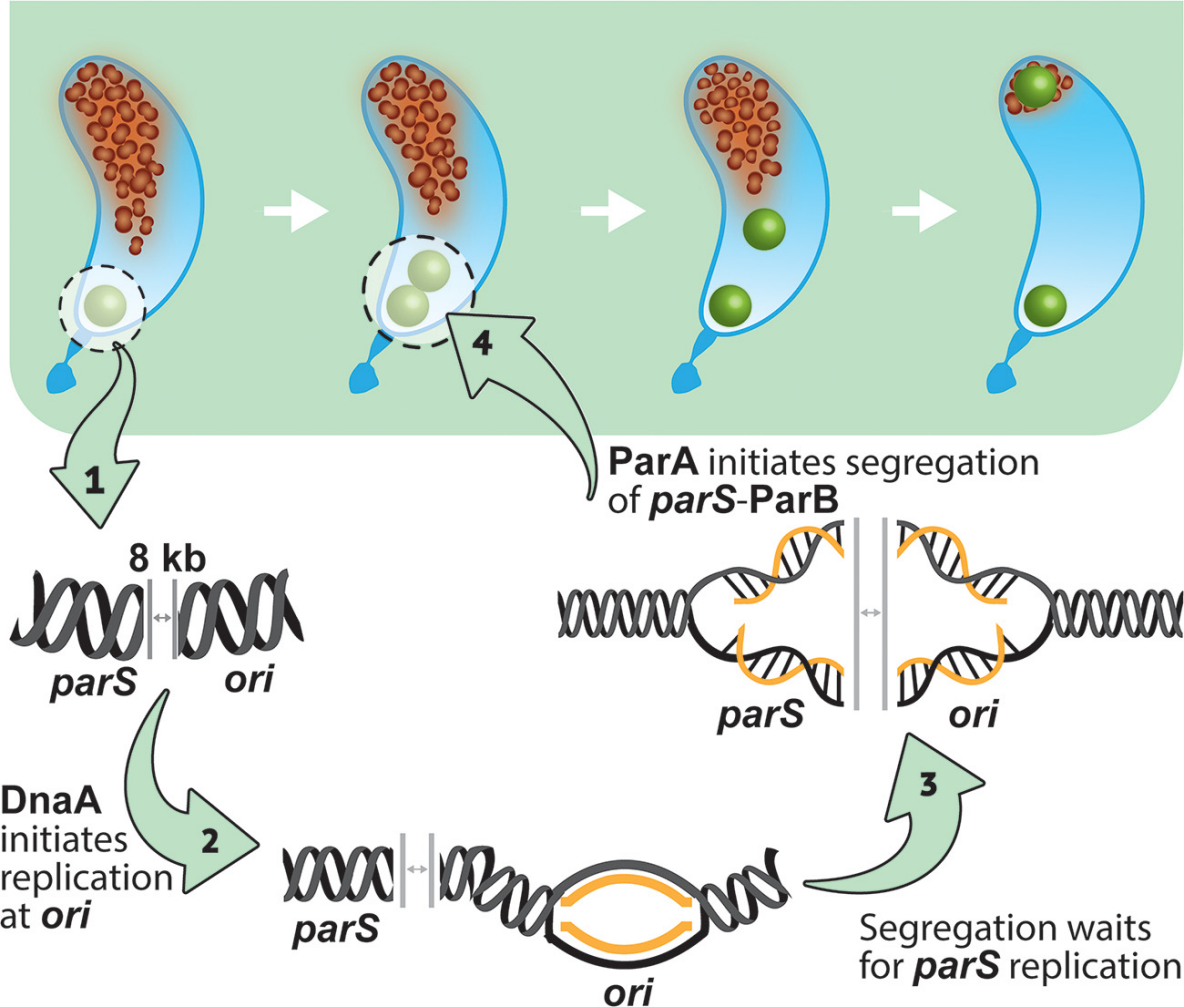
Schematic of C. crescentus asymmetric cell cycle, highlighting the dynamics of the onset of chromosome replication and segregation. Nondividing cells contain the origin of replication (*ori*) and the centromere-like region (*parS*) (green dot) near the stalked pole. The origin of replication and the ParB/*parS* complex segregate first toward the new pole in a ParA-dependent fashion, following the cloud-like gradient of ParA retracting (top panel). DnaA initiates chromosome replication at *ori*. ParA does not initiate chromosome segregation until *parS* is replicated (bottom panel).

In C. crescentus, the ParABS system is necessary for chromosome segregation, and, thus, essential for viability (19). The asymmetric cell cycle of C. crescentus has facilitated extensive work on the ParABS system, revealing fine details of the connection between the onset of replication and segregation. For instance, once replication initiates at *ori*, chromosome segregation is not triggered until the *parS* chromosomal locus is replicated (20) (Fig. 1). The relatively high concentrations of ParB molecules bound at *parS* are necessary to trigger the ATPase activity of ParA (21). Dimers of ParA interact with the chromosome nonspecifically, forming a cellular gradient that retracts as 1 copy of the replicated *parS* (decorated with ParB) segregates to the opposite pole (22–24) (Fig. 1). Connections between the onset of replication and segregation have been proposed in C. crescentus. For instance, DnaA, aside from binding *ori*, was shown to bind *parS* directly and promote the translocation of *parS*-ParB to the opposite pole in a ParA-dependent manner (25). Interestingly, the nucleoid-associated protein GapR, similarly to DnaA, binds at *ori* and *parS*, coordinating the onset of chromosome replication with the asymmetric chromosome separation (26). Although C. crescentus serves as a powerful system to investigate the coordination between the onset of chromosome replication and the onset of segregation, the potential role of ParA as a modulator of chromosome replication initiation has not yet been characterized.

In this study, we discovered that ParA in C. crescentus is also connected to the onset of chromosome replication, likely through an indirect mechanism. We demonstrate that ParA levels can significantly alter the number of *ori* per cell in a DnaA-ATP dependent manner. ParA was unable to promote replication initiation in cells with high levels of the DnaA-deactivator HdaA, suggesting that ParA is not involved in DnaA’s nucleotide activation step. Using a set of ParA variants, we demonstrate that cells accumulate multiple *ori* regions, regardless of whether the ParA levels increased are of variants unable to promote the translocation of *parS-*ParB to the opposite pole. Our data also revealed that increased levels of ParA increased the ability of suboptimal levels of DnaA to initiate chromosome replication. Collectively, these data provide new insights into the complex coordination between these 2 fundamental events involved in the maintenance of chromosome integrity.

## RESULTS

### ParA perturbs frequencies of chromosome replication initiation.

**FIG 2:**
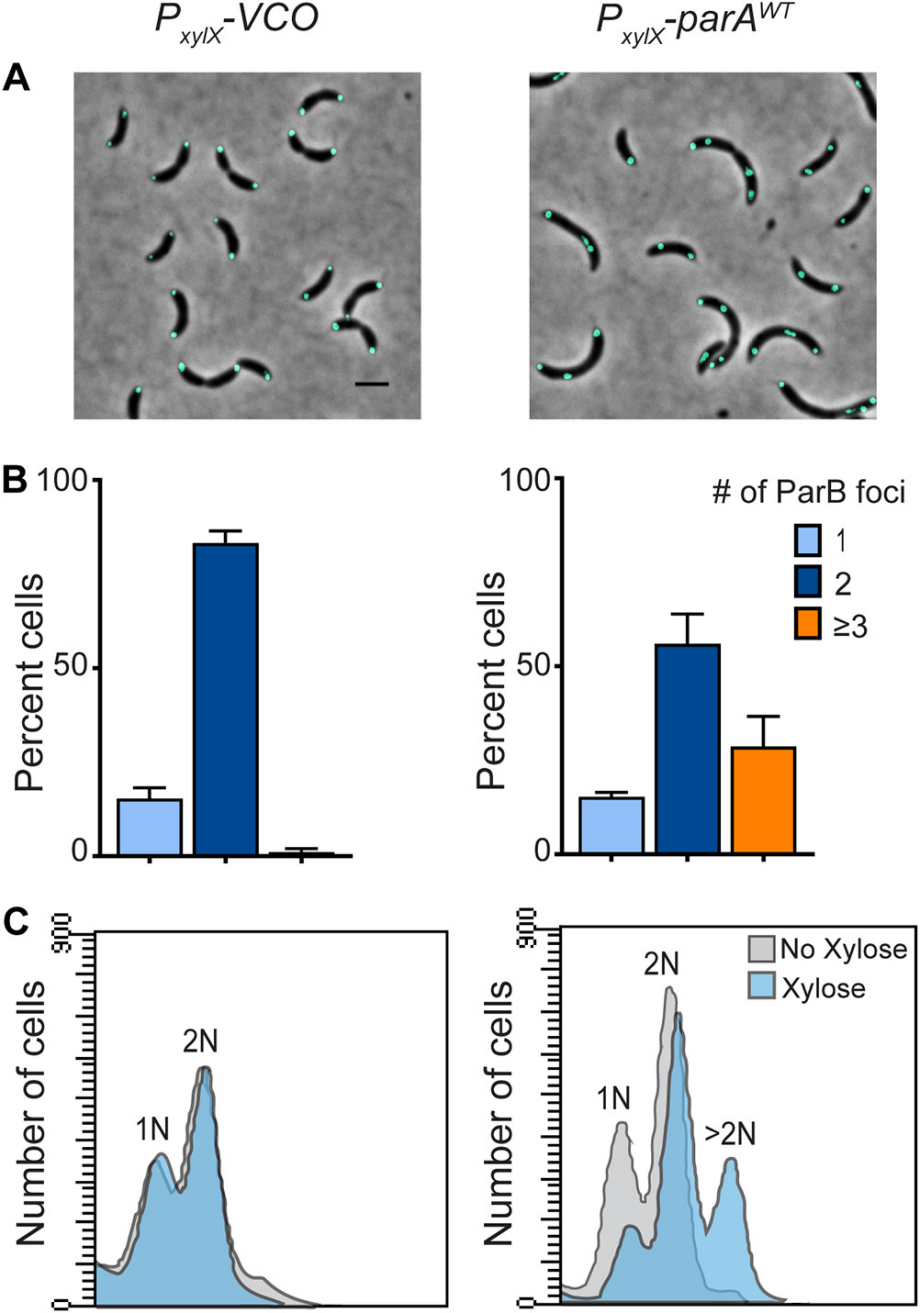
High levels of ParA^WT^ promote *ori* supernumerary in C. crescentus. (A) Micrographs of CB15N, *parB::cfp-parB* cells with empty vector control, and *xylX::parA^WT^* expressing ParA^WT^ for 3 h. Green foci - CFP-ParB. (B) Bar graphs of the percent number of ParB foci (*ori*s) per cell. (C) Flow cytometry profiles of cells after 3 h of incubation. Micrograph scale bar −2 μm. Data represents 3 independent experiments with error bars of mean ± standard deviation (SD).

We previously reported an intriguing observation where cells overexpressing *parA* seemed to be able to initiate chromosome replication in cells depleted of the replication initiator DnaA (27). We hypothesized then that ParA could potentially be promoting this initiation of chromosome replication. In this study, we tested this initial hypothesis by analyzing the frequency of replication initiation in wildtype cells overexpressing *parA*. To do that, we constructed a merodiploid C. crescentus strain expressing *parA* from the chromosomal xylose inducible promoter in addition to the native copy of *parA* under its own promoter (PM541: NA1000, *xylX::parA*, *parB::cfp-parB*). To track the initiation of chromosome replication, we fluorescently labeled the partitioning protein ParB (CFP-ParB) that binds the centromere-like region *parS* (28). Because the *parS* locus is found near the origin of replication (20), we can infer the frequency of replication initiation by counting the number of CFP-ParB foci per cell (28). Our data revealed that cells (PM541) overexpressing *parA* display multiple CFP-ParB foci, suggesting that these cells were re-initiating chromosome replication potentially due to increased levels of ParA (Fig. 2A). The addition of the same xylose concentrations to a strain with an empty-vector control (PM566: NA1000, *xylX*::empty-vector, *parB::cfp-parB*) resulted in cells behaving like the wild-type control, displaying only 1 or a maximum of 2 CFP-ParB foci per cell (Fig. 2B).

We used 2 additional methods to confirm the impact of *parA* overexpression on replication initiation. Firstly, we quantified the number of *ori* independent of ParB by testing a C. crescentus strain, in which the area near the origin of replication (at CCNA_00006) is fluorescently labeled with TetR-eYFP and a *tetO* array (29). Consistent with our ParB-labeled cells, increasing the levels of *parA* expression in this *ori* labeled strain resulted in multiple *ori* foci (Fig. S1A and B). Secondly, we quantified the chromosomal content of cells overexpressing *parA* using flow cytometry. Cells that were induced for *parA* overexpression displayed an additional curve of >2 chromosomes per cell (Fig. 2C). This increase in chromosomal number was not observed in cells with vector control only (VCO) treatment under the same conditions. These cumulative data confirmed that increasing levels of *parA* expression results in cells comprising of multiple origins of replication.

Furthermore, we determined the levels of ParA that were able to accumulate supernumerary *ori* regions using our *parA* merodiploid strain. To track the over-expression levels of *parA*, the protein ParA was tagged with the peptide M2 (DYKDDDDK) at the C-terminus. We first confirmed that the expression of ParA-M2 from *parA*’s native promoter had no effects on growth (Fig. S2A). Cells expressing *parA-M2* from the xylose promoter retained their ability to trigger the supernumerary *ori* regions (Fig. S2B). To determine the levels of ParA, we compared native *parA-M2* expression levels versus *xylX::parA-M2* expression using Western blots. Our data revealed that overexpression of *parA* from the xylose promoter results in an ~5-fold increase of ParA levels after 3 h of induction compared to VCO (Fig. S2C and D).

### Supernumerary *ori* regions is triggered independently from ParA’s cloud localization.

**FIG 3:**
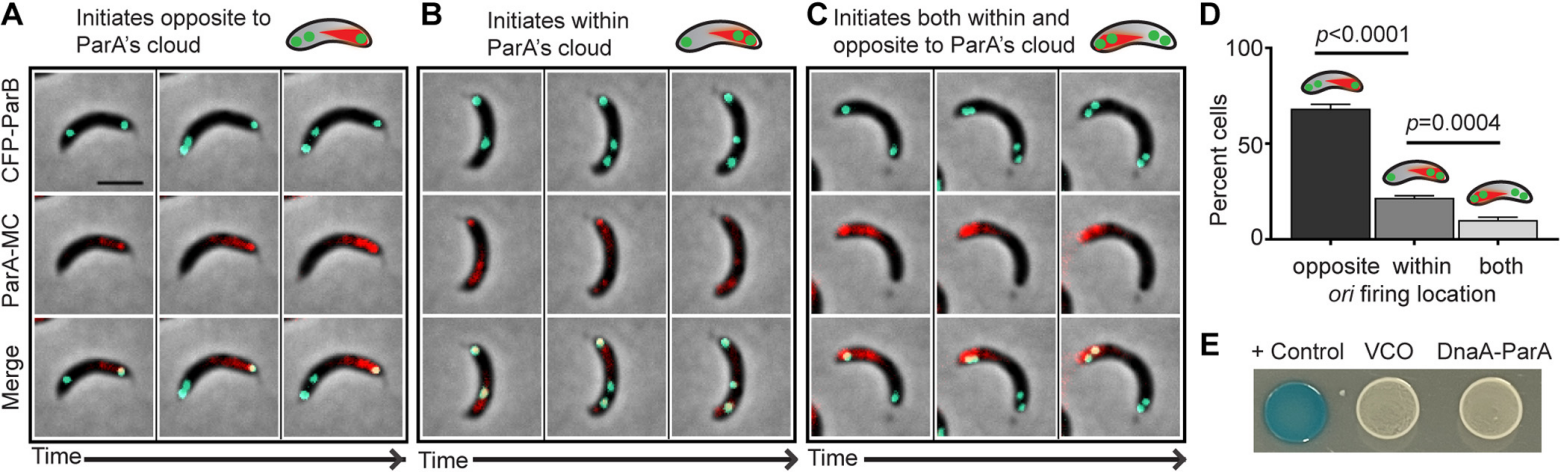
Replication re-initiates independent of ParA’s cloud localization. Phase contrast fluorescence micrographs of CB15N *parB::cfp*-*parB*, *xylX::parA-mCherry* (MC) cells expressing ParA^WT^-MC. Swarmer cells were incubated with xylose (0.1%) for 75 min, and spotted (*t* = 0 h) on a M2G agar pad containing xylose (0.1%). Time lapse micrographs were obtained every 15 min over 150 min. Cells in (A) showing the initiation of replication opposite to, (B) within and (C) both within and opposite to, ParA’s cloud. Time points: (A) 30, 60, and 90 min, (B and C) 45, 75, and 105 min. Culture (OD_600_ 0.3) was supplemented with xylose (0.1%) prior to synchrony to separate swarmer cells. Micrographs data are representative of 3 independent experiments. Micrograph scale bar −2 μm. (D) Quantification of *ori* firing events. Bar graph data are from 3 independent experiments with error bars of mean ± SD, one-way ANOVA statistical analyses, and (E) bacterial two-hybrid assay. DHM1 cells of + control with pKT25-zip/pUT18C-zip plasmids, vector control (VCO) with pKT25/pUT18C plasmids, and test with pUT18-ParA/pKNT25-DnaA plasmids on LB agar plates containing X-gal/IPTG.

In B. subtilis, the monomeric form of Soj (ParA) has been shown to directly interact with DnaA and inhibit DnaA’s ability to initiate chromosome replication (17). To determine whether ParA in C. crescentus could interact with DnaA, we analyzed the localization of DnaA and ParA prior to chromosome replication re-initiation. In C. crescentus, cells expressing a fluorescently labeled ParA display a cloud-like structure that retracts during *parS* segregation (Fig. 1) (22, 24, 30). This cloud-like structure and its retraction during segregation were observed in cells expressing *parA* fluorescently labeled from the native promoter, or expressed from inducible promoters in merodiploid *parA* strains (22–24). To investigate whether the over-initiation of replication occurred within or away from ParA’s cloud, we fluorescently labeled ParA (ParA-mCherry) in a *parA* merodiploid strain, and analyzed its localization patterns. We determined where replication re-initiation occurs (where *ori-parS* localizes) inside the cell in reference to ParA’s gradient. To do this, we performed a timelapse to track before and after the appearance of multiple ParB foci. Our data suggest that ParA’s impact on replication initiation can occur independently from the spatial organization of *ori-parS* and ParA’s cloud. Although re-initiation of replication occurs at a higher frequency when *ori* is located away from ParA’s cloud (68%), we also observed cells that re-initiated replication when *ori* was found within ParA’s cloud (22%), or in the same cell within and away from ParA’s cloud (10%) (Fig. 3A to D). One limitation of this analysis is that the merodiploid strain used in these experiments has the native *parA* expressed in the background with no fluorescent tag, which limits potential interpretations of the amount of ParA associated with ParB foci upon initiation of replication. To further analyze the possibility of direct protein-protein interactions, we performed a bacterial two-hybrid (B2H) system. The B2H analysis revealed negative results, suggesting that, under the conditions tested, DnaA and ParA may not directly interact (Fig. 3E). Further analyses are necessary to conclusively determine whether DnaA and ParA interact.

### ParA’s impact on replication initiation is DnaA-dependent.

**FIG 4:**
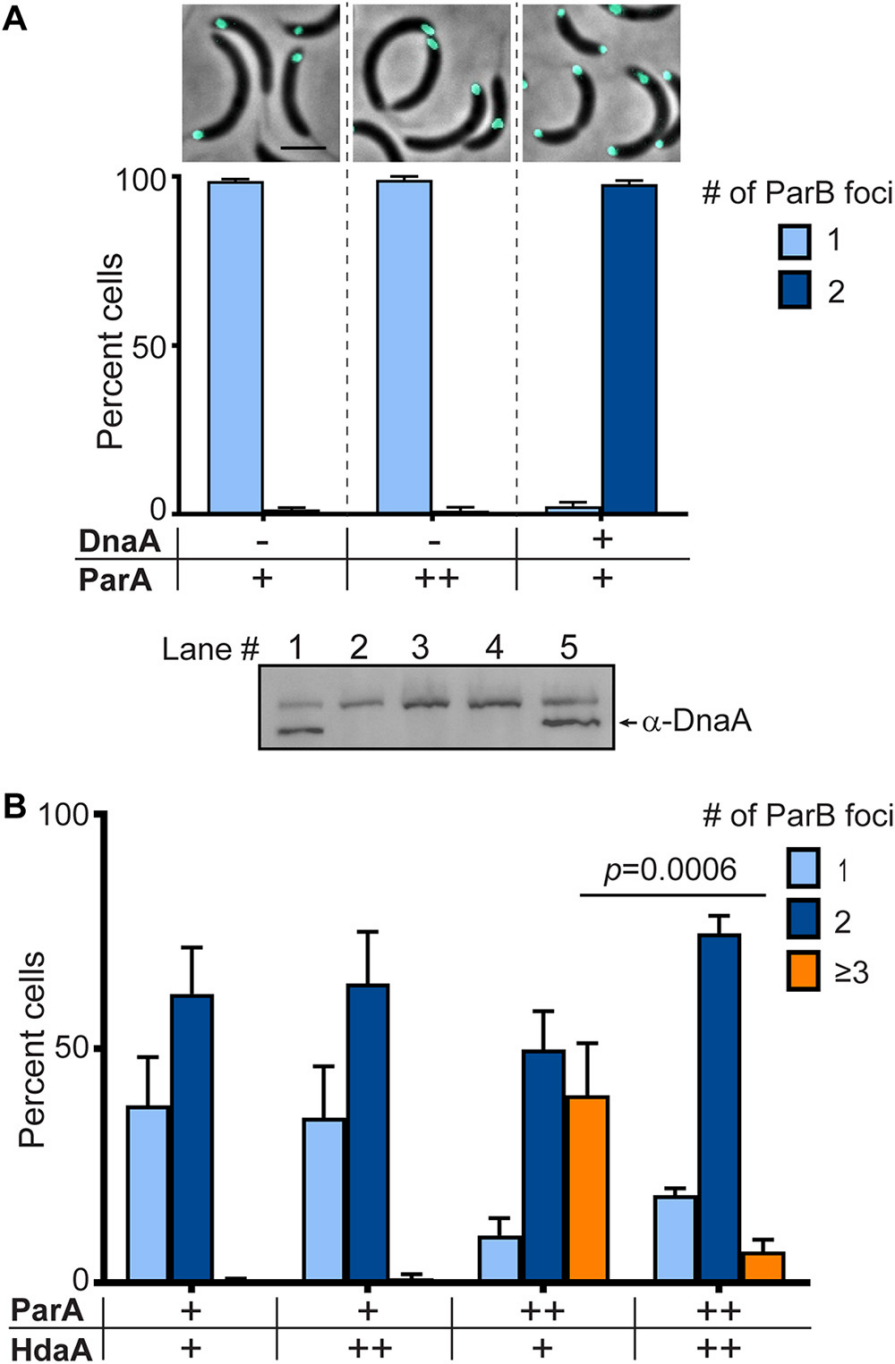
DnaA-ATP is required for ParA to promote replication initiation. (A) Micrographs of C. crescentus CB15N, *parB::cfp*-*parB*, Δ*vanA*, *dnaA::*Ω, *vanA::dnaA*, and *xylX::parA^WT^* cells, and quantification plots of the number of CFP-ParB foci/*ori* per cell: depleted for 4 h (left), 3 h DnaA depleted cells expressing ParA^WT^ under xylose promoter for 1 h (middle), and 3 h DnaA depleted cells expressing DnaA for 1 h (right). Western blot (bottom) of DnaA levels of each cell sample: equal amount of cells loaded onto lanes: 1, swarmer cells; 2, DnaA depleted cells for 3 h; 3, DnaA depleted cells for 4 h; 4, 3 h DnaA depleted cells expressing ParA^WT^ for 1 h; and 5.3 h DnaA depleted cells expressing DnaA for 1 h. (B) Bar graph of CFP-ParB foci quantification of merodiploid ParA and HdaA (CB15N, *parB::cfp*-*parB*, Δ*vanA*, *vanA::*hda*A*, *xylX::parA^WT^*) cells expressing HdaA and/or ParA. Micrograph scale bar −2 μm, (+) wild-type gene expression, and (++) induced protein levels under wild-type background. Bar graph data are from 3 independent experiments with error bars of mean ± SD and two-way ANOVA statistical analyses.

Given that increased levels of ParA result in cells with >2 chromosome equivalents, we tested the possibility of ParA promoting the onset of chromosome replication independently of the canonical replication initiator DnaA. Because DnaA is essential for viability, we tested this DnaA-independent replication hypothesis using a strain where the levels of DnaA can be depleted. This depletion is accomplished by replacing the native *dnaA* copy with a streptomycin cassette, and incorporating a *dnaA* copy downstream of the inducible chromosomal promoter vanillate (van) (PM109) (25, 31). We engineered into PM109 a second copy of *parA* under the inducible promoter xylose (PM542: *dnaA::spec*, *vanA::dnaA*, *xylX::parA*, *parB::cfp-parB*). These cells (PM542) were depleted of DnaA for 3 h, and then induced for *parA* overexpression for an additional hour. Cells depleted of DnaA displayed only 1 CFP-ParB focus, consistent with their inability to initiate chromosome replication in the absence of DnaA (Fig. 4A). In our positive control condition, ~98% of cells that had DnaA depleted (3 h), and then repleted (1 h), displayed 2 CFP-ParB foci, showing that DnaA has no problem initiating replication after the depletion period. In the potential scenario where ParA can initiate chromosome replication independently of DnaA, PM542 cells depleted of DnaA and then exposed to xylose to induce *parA* expression would also display 2 CFP-ParB foci per cell. However, our data revealed that cells overexpressing *parA* (+ xylose) in the absence of DnaA (no van) display only 1 CFP-ParB focus per cell (Fig. 4A). High levels of ParA had no effect on the number of CFP-ParB foci in cells that lacked DnaA. These data indicate that ParA cannot initiate chromosome replication on its own. ParA’s apparent role on replication initiation is DnaA-dependent, although not necessarily via direct DnaA-ParA interaction.

We examined another possible scenario where ParA stimulates replication initiation with deactivated DnaA (ADP-bound DnaA). To test this hypothesis, we utilized the replisome-associated Hda protein involved in the Regulatory Inactivation of DnaA (referred to as RIDA) (4). Hda is a conserved DnaA-related protein that converts active DnaA (ATP-bound DnaA) to deactivated DnaA (ADP-bound DnaA) (32, 33). In C. crescentus, depletion of Hda (known as HdaA) levels results in over-initiation of replication, whereas overexpression of *hdaA* results in replication initiation delays (29, 34). To determine whether HdaA had any effect on ParA’s ability to promote replication initiation, we characterized cells with high levels of HdaA. We constructed a merodiploid *hdaA* strain with the additional copy of *hdaA* under the chromosomal van inducible promoter (PM607: *vanA::hdaA*, *xylX::parA*, *parB::cfp-parB*). PM607 cells were pre-induced for overexpression of *hdaA* for 1 h. Post *hdaA* induction, the frequency of replication initiation was quantified. In our control sample, cells overexpressing *parA* alone displayed multiple *ori* foci in about 40% of cells. However, when the expression of *hdaA* was co-induced with *parA*, the number of cells displaying multiple *ori* dropped significantly (Fig. 4B). These data revealed that high levels of HdaA can prevent ParA’s impact on the onset of chromosome replication.

### ParA promotes the initiation of replication at sub-optimal levels of DnaA.

**FIG 5:**
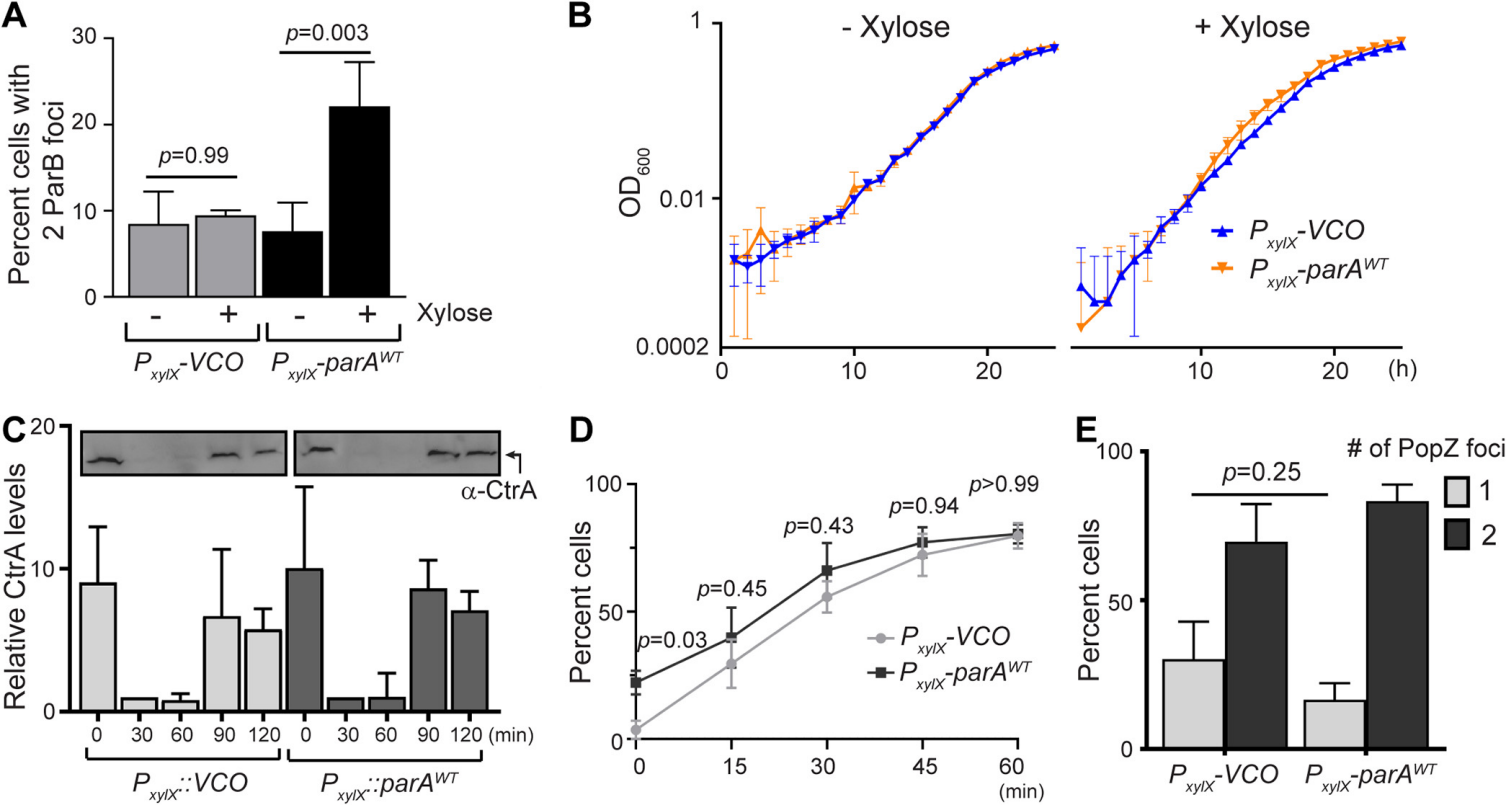
Changes in replication initiation result in unaltered growth rate. (A) ParA promotes the initiation of replication at sub-optimal levels of DnaA. Plot of number of cells with 2 CFP-ParB foci/*ori* of CB15N, *parB::cfp*-*parB*, Δ*vanA*, *dnaA::*Ω, *vanA::dnaA*, *xylX::empty-vector*, and *xylX::parA^WT^* expressing ParA^WT^ for 1 h with vanillate (0.5 μM) and ± xylose (0.1%) after depleting DnaA for 2 h. (B) High levels of ParA promotes replication initiation without disturbing growth. Growth curves of C. crescentus CB15N, *parB::cfp-parB* cells with empty vector control, and *xylX::parA^WT^* in minimal (M2G) medium ± 0.1% xylose. (C) Bar graph of relative levels of CtrA expression in cells with VCO and higher levels of ParA^WT^ in M2G media supplemented with xylose (0.1%). Cells grown in M2G media were supplemented with xylose (0.1%) 1 h prior to synchrony to induce ParA expression. Swarmer cells in M2G with xylose (0.1%) were incubated at 30°C in a roller-shaker for 2 h. Cell pellets (normalized to OD_600_ 0.2) were saved at *t* = 0, 30, 60, 90, and 120 min for Western blots. (D) Quantification plots of percent cells that contain 2 CFP-ParB foci in cells expressing an empty vector control (VCO) or *xylX::parAWT*. Cells were pre-induced with 0.1% xylose for 1 h, synchronized, then placed on an agar pad containing 0.1% xylose. Phase contrast fluorescence micrographs were imaged every 15 min, up to 1 h. (E) Bar graphs of the percent number of PopZ-YFP foci of CB15N, *popZ::popZ-yfp* cells with empty vector control, and *xylX::parA^WT^* expressing ParA^WT^ for 3 h. (A to D) Data are from 3, and (E) from 4, independent experiments with error bars of mean ± SD and two-way ANOVA statistical analyses.

Given that ParA was unable to promote replication initiation in the absence of DnaA, we analyzed the potential ability of ParA to promote chromosome replication initiation in cells expressing sub-optimal levels of DnaA. We first determined the minimum induction levels necessary for DnaA to initiate chromosome replication (Fig. S3). To do that, we used strain PM542 (*dnaA::spec*, *vanA::dnaA*, *xylX::parA*, *parB::cfp-parB*) to deplete DnaA levels first, and then titrate back van to induce different levels of *dnaA* expression. The concentration of van typically used to induce expression of the van promoter is 250 μM (35). Within 1 h of induction (post-depletion), cells expressing *dnaA* from the P_van_ promoter at 10 μM van displayed the same frequency of replication initiation as 250 μM van treatment. Van concentrations of 0.5 μM or below were insufficient to trigger replication initiation after 1 h induction (Fig. S3). Using 0.5 μM van, we tested whether high levels of ParA could influence the ability of these cells to initiate replication. Our xylose-VCO strain was depleted of DnaA and then exposed to xylose, displaying only 1 ParB focus per cell, demonstrating that the addition of xylose has no impact on the onset of chromosome replication under the conditions tested (Fig. 5A). Notably, when cells contain sub-optimal levels of DnaA and high levels of ParA, the frequency of chromosome replication initiation increases, as evidenced by the higher number of cells displaying 2 CFP-ParB foci. These data confirm our previous hypothesis that *parA* overexpression results in cells able to initiate replication at sub-optimal DnaA levels (27).

### Cells with supernumerary *ori* regions display unaltered growth rate.

E. coli is known to initiate multiple rounds of replication when grown in rich media, allowing it to complete a cell cycle in less time than the time that is necessary to complete the replication of its chromosome (36). Unlike E. coli, C. crescentus initiates replication only once per cell cycle, regardless of the media type (37). Given that cells with high ParA levels were initiating multiple rounds of replication per cell cycle, we tested whether C. crescentus can double in shorter times in cells overexpressing *parA*. Using growth curves, our data revealed that the increased frequency of replication initiation has no impact on the doubling time compared to our wild-type control (Fig. 5B).

We next investigated whether the overall progression of the cell cycle was altered when ParA levels were increased using 2 indicators of C. crescentus: the master cell cycle regulator CtrA, and the chromosomal anchoring protein PopZ (38, 39). The levels of CtrA are tightly regulated at multiple levels over the cell cycle (40). Our data revealed that the cell cycle profile of CtrA levels remained the same in cells overexpressing *parA*, just like the vector-only control (Fig. 5C). Although CtrA levels remained unchanged, we questioned whether high levels of ParA could alter CtrA’s ability to inhibit replication initiation in swarmer cells (29, 41). To test this hypothesis, we quantified the timing of onset of chromosome replication using synchronized cells that were pre-induced for *parA* overexpression (Fig. 5D). We reasoned that if ParA could alter CtrA’s ability to inhibit replication, cells would start initiating chromosome replication earlier than wildtype cells. Our data revealed that overexpressing *parA* results in a small, but significant, increase in cells with 2 *ori* only at time zero with no significant differences in the latter time points compared to VCO. The small number of cells that display 2 *ori* at time zero could potentially have arisen from cells that were already born with 2 *ori* from mother cells containing supernumerary *ori*s. To determine whether potential deregulation of anchoring/translocation of the *parS* locus caused the supernumerary *ori* regions, we tracked PopZ localization using a strain expressing a fluorescently labeled *popZ* from the native promoter (42). Our analyses revealed that PopZ localization at the cell poles is not altered by the overexpression of *parA* (Fig. 5E). Collectively, our data suggest that ParA’s impact on chromosome replication is not attributed to defects in CtrA’s regulation of chromosome replication, nor to defects of chromosome anchoring at the cell poles.

### Expression of segregation-deficient ParA variants results in cells with multiple *ori* regions.

**FIG 6:**
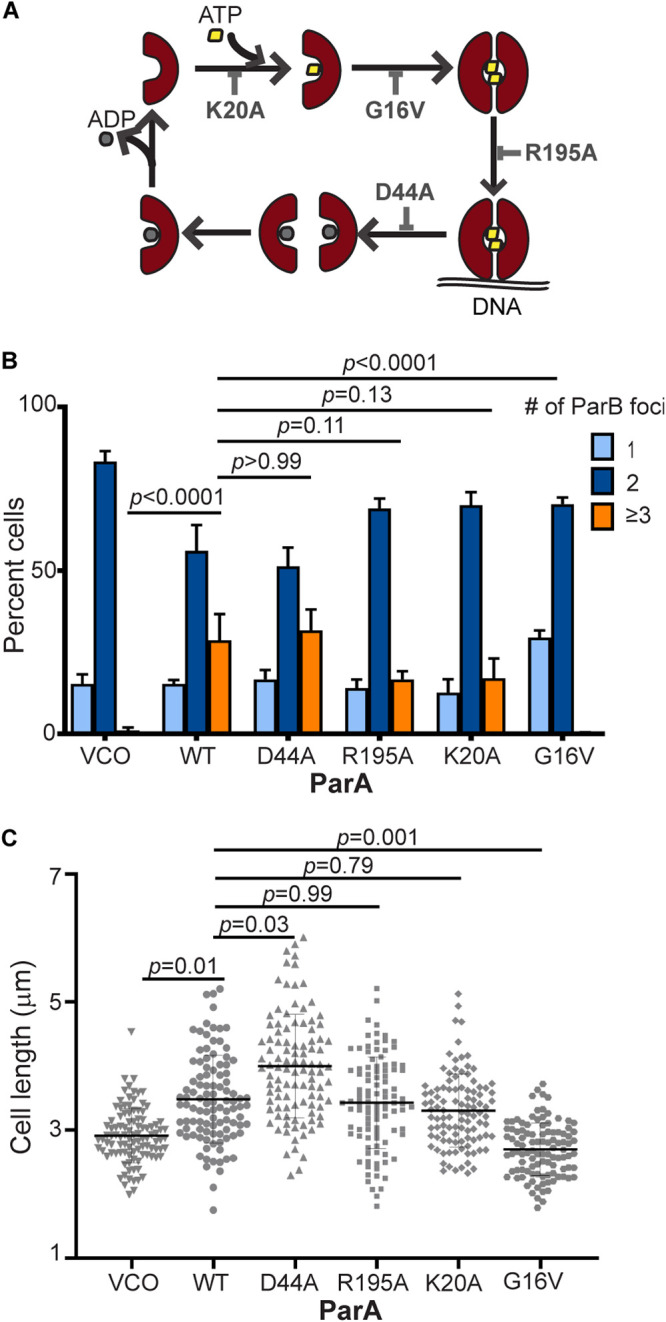
ParA levels are linked to initiation of replication and cell length regulation. (A) Cartoon representation of the ParA cycle (21). (B) Quantification plots of percent cells of CFP-ParB foci in cells expressing ParA variants: empty vector control (VCO), *xylX::parA^WT^*, *xylX::parA^D44A^*, *xylX::parA^R195A^*, *xylX::parA^K20A^*, and *xylX::parA^G16V^* for 3 h. Swarmer cells of ParA variants were incubated in M2G media supplemented with xylose (0.1%) for 3 h, and phase contrast fluorescence micrographs were imaged. Data are from 3 independent experiments with error bars of mean ± SD and two-way ANOVA statistical analyses. (C) Scatter dot plot of cell length of ParA variants in the presence of xylose (0.1%) for 3 h. Mix population of ParA variants were incubated at 30°C for 3 h in M2G media with xylose (0.1%) before obtaining micrographs. ns represents not significant. Data are from 3 independent experiments with error bars of mean ± SD and Nested *t* test statistical analyses.

To explore the potential mechanism involved in ParA’s ability to promote the onset of replication, we analyzed a set of ParA variants that disturb its protein dynamics (Fig. 6A). Given that ParA is essential for chromosome segregation (19, 20), the expression of each *parA* variant was regulated by the chromosomal xylose promoter in an otherwise wild-type background. These ParA variants have been previously shown to express stable proteins in C. crescentus (22, 30). To determine whether ParA can promote replication initiation independent of its ability to promote chromosome segregation, we analyzed 2 ParA variants that can form dimers but are inactive in chromosome segregation. Firstly, we used the dominant negative ParA^D44A^ variant that can bind ATP and dimerize, but it is unable to hydrolyze ATP (15, 22, 43). In other words, ParA^D44A^ is trapped as ParA dimers bound to ATP. Expressing this dimer variant ParA^D44A^ from the xylose promoter resulted in *ori* supernumerary frequency that was similar to that observed in cells over-expressing wildtype ParA (ParA^WT^) (Fig. 6B). We next examined the role of ParA’s ability to bind DNA and its impact on replication initiation. The ability of dimers of ParA to interact with DNA nonspecifically is essential for ParA’s role in chromosome segregation in C. crescentus (19, 20). We focused on the conserved residue Arg195, previously shown to be critical for ParA’s ability to bind DNA (30, 44). ParA^R195A^ can bind ATP and dimerize, but is unable to interact with DNA (45). Our data revealed that cells expressing *parA-*(*R195A*) displayed multiple CFP-ParB foci. One caveat of this analysis is that the native *parA* is expressed in the background, which could result in the formation of heterodimers. The heterodimers ParA^R195A^-ParA^WT^ could potentially retain the ability to bind DNA, limiting our ability to conclude on the role of DNA binding. Our data with the dominant negative variant ParA^D44A^ suggest that the effect on the onset of chromosome replication does not require ParA’s ability to trigger chromosome segregation.

### The monomer ParA-ATP does not impact the onset of chromosome replication.

Given that cells with increased levels of ParA dimers unable to promote chromosome segregation also accumulate multiple *ori* regions, we examined the effect of monomers of ParA and their impact on replication initiation. We focused on 2 forms of ParA monomers: apo-ParA and ParA bound to ATP (ParA-ATP). To examine the apo-ParA, we focused on the conserved Lys20 residue located in the Walker A motif critical for nucleotide binding (43). The change of Lys to Ala in this residue has been shown to prevent ParA’s ability to bind ATP, and is therefore unable to dimerize (30). Like wildtype ParA, cells expressing *parA*-(K20A) also displayed *ori* supernumerary (Fig. 6B). Because our construct strain has the ParA^WT^ in the background, we could not conclude definitively that ParA^K20A^ remains as a monomer under the conditions tested. A potential scenario is that ParA^K20A^ could form heterodimers with ParA^WT^. Thus, instead of using nucleotide binding, we next used a ParA variant that is unable to dimerize because of steric clashes. To do that, we analyzed cells expressing the variant encoding ParA^G16V^, and quantified the frequencies of over-initiation of chromosome replication. The glycine to valine change (G16V) renders ParA able to bind ATP, but unable to form dimers due to steric clashes (43). Unlike the over-expression of wild-type ParA, cells expressing *parA*-(G16V) did not display multiple *ori* regions. Cells expressing *parA*-(G16V) displayed only 1 or a maximum of 2 CFP-ParB foci per cell (Fig. 6B). We confirmed that *parA-*(G16V) was being expressed in these cells with 1 or 2 CFP-ParB foci by examining a fluorescently labeled version of this variant (Fig. S4). Unlike the fluorescently labeled, wildtype ParA, cells expressing the fluorescently labeled ParA^G16V^ were unable to over-initiate chromosome replication.

**FIG 7:**
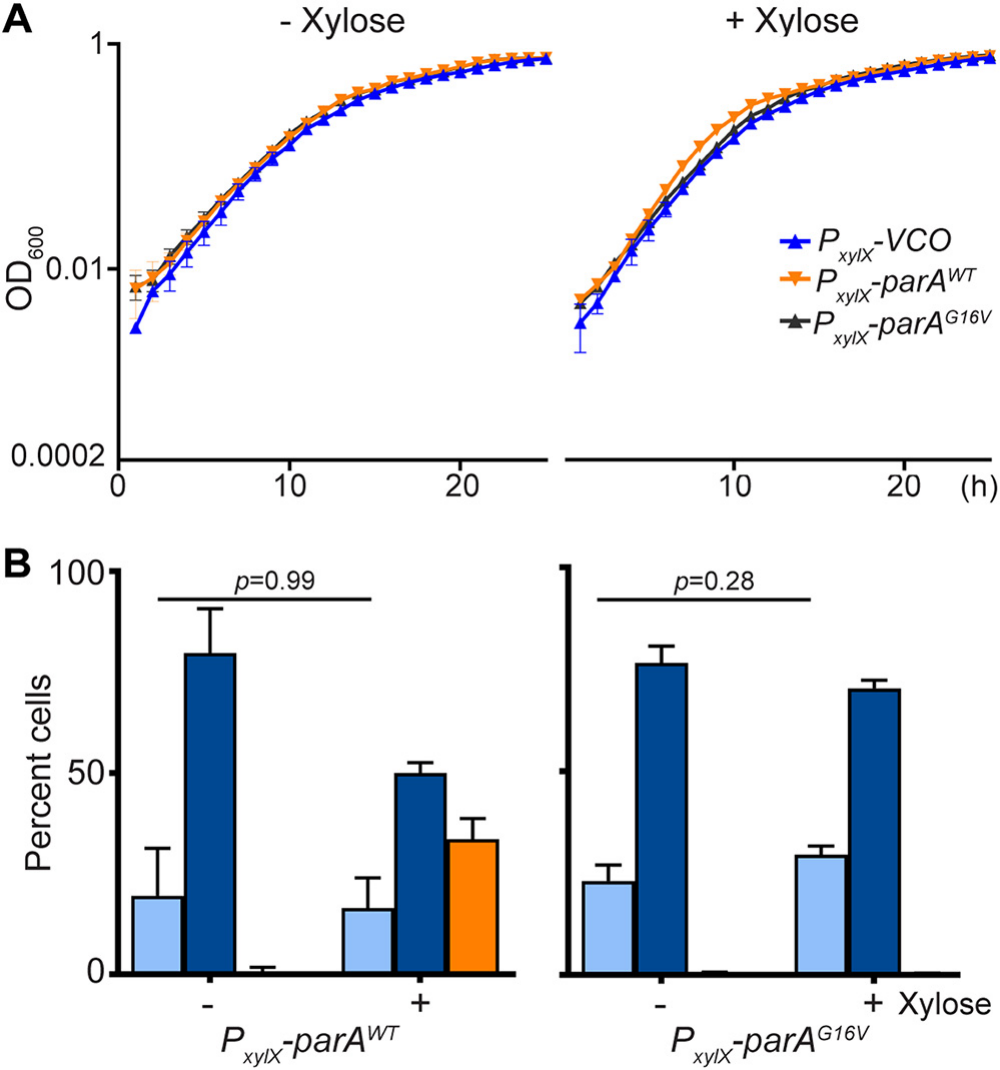
ParA^G16V^ displays no impact on replication initiation in C. crescentus. (A) High levels of ParA^G16V^ do not alter the growth rate. Growth curves of C. crescentus CB15N, *parB::cfp-parB* cells with empty vector control, *xylX::parA^WT^*, and *xylX::parA^G16V^* in minimal (M2G) medium ± 0.1% xylose. (B) Quantification plots of percent cells of CFP-ParB foci in cells expressing ParA variants *xylX::parA^WT^* and *xylX::parA^G16V^* for 3 h. Swarmer cells of ParA variants were incubated in M2G media supplemented with xylose (0.1%) for 3 h and phase contrast fluoresce micrographs were imaged. Data are from 3 independent experiments with error bars of mean ± SD and two-way ANOVA statistical analyses.

Because the monomer of Soj (ParA) has been shown to inhibit replication initiation by directly binding DnaA (17), we further characterized the potential inhibitory effect in C. crescentus. We reasoned that, if the expression of *parA*-G16V is inhibitory, a lower frequency of replication initiation would impact the cells’ doubling time. To test this hypothesis, we performed growth curves of cells expressing *parA*-G16V compared to empty-vector control. The growth curve analyses revealed no significant differences in the doubling times in cells with or without ParA^G16V^ (Fig. 7A). Furthermore, we compared the ability of cells to initiate replication when grown with and without inducer for *parA*-G16V expression. Increasing the levels of ParA^WT^ resulted in an increased rate of cells with multiple *ori* (Fig. 7B). In the scenario where ParA^G16V^ inhibits replication initiation, we would expect to find a higher number of cells expressing *parA*-G16V with a single *ori*. However, cells with or without inducer for *parA*-G16V displayed the same number of cells with 1 or 2 *ori* (Fig. 7B). Collectively, our data revealed that the monomer ParA^G16V^ cannot inhibit chromosome replication initiation. However, we cannot conclude that ParA in C. crescentus does not play an inhibitory role of replication initiation, as has been observed in other bacteria. Our results suggest that, if there is an inhibitory effect on replication initiation, the monomer ParA does not seem to be the one involved in the direct inhibition.

### ParA levels are linked to the initiation of replication and cell length regulation.

In E. coli, the over-initiation of replication results in cells with longer cell length (46). However, mechanistic details about this connection including information about which event causes which (over-initiation of replication versus cell length) have remained elusive in the field. We examined the impact on cell length and *ori* supernumerary in C. crescentus (Fig. 6C). Our data revealed that increasing levels of wildtype ParA, which results in multiple (> 2) copies of *ori*, also increases cell length. The cell length remained the same as the VCO in cells expressing the variant ParA^G16V^, which cannot promote the over-initiation of replication. Like in E. coli, these data revealed a similar connection between cell length and over-initiation of chromosome replication in C. crescentus. However, cells expressing the dimer variant ParA^D44A^ displayed an even higher increase in cell length compared to ParA^WT^ (Fig. 6B), while maintaining the same frequency of *ori* supernumerary (Fig. 6C). These data suggest that different forms of ParA may impact cell length independently from chromosome replication initiation.

Aside from cells becoming longer, overexpression of *parA* for 6 h in rich media has previously been shown to result in the release of anucleated mini-cells in C. crescentus (19). To determine whether the *ori* supernumerary was caused by the release of mini-cells, we quantified the frequency of mini-cells present under our experimental conditions of 3 h induction of *parA* expression grown in minimal media (Fig. S5). Our data revealed that in cells that display ~25% of *ori* supernumerary, only 2.6% of mini-cells were present at that time point. Although we cannot eliminate the possibility that cell length has a partial role in the observed over-initiation of replication, our data revealed that mini-cell formation is unlikely a contributor to the deregulation of replication in cells with *parA* overexpressed.

## DISCUSSION

Successful generation of a daughter cell requires the complex coordination of multiple events for proper progression of the cell cycle. The goal of this study was to identify the role of the partitioning protein ParA in coordinating other cell cycle events in C. crescentus. Our data revealed that cells overexpressing *parA* display supernumerary *ori* regions. ParA can promote replication initiation in cells expressing sub-physiological levels of the replication initiator protein DnaA that are not sufficient to initiate replication on their own. We show that the ability of ParA to impact chromosome replication initiation also occurs in cells expressing variants of ParA unable to promote chromosome segregation.

Based on our analyses, ParA’s impact on replication initiation in C. crescentus is likely an indirect result from altered cell cycle events. For instance, ParA could affect chromosome replication initiation by altering other regulators of the cell cycle network, like regulators that bind at or near *ori*. One obvious player was CtrA because it inhibits replication initiation by outcompeting DnaA for binding *ori* (29, 41). We show that high levels of ParA do not impact growth rate, nor the tightly regulated CtrA levels. These data are consistent with previous analyses where changes in levels of the par system resulted in no changes in the localization of CtrA’s regulator (CckA), nor changes in expression levels of genes regulated by CtrA (47). Another possible factor is ParB, which binds the centromere-like region *parS* and loads the Structural Maintenance Complex (SMC) for proper chromosomal arm alignment (20, 48). One potential scenario is that high levels of ParA compete for ParB’s binding with SMC resulting in differences in alignment of the chromosomal arms near *ori*. Using the genome-wide chromosome conformation capture assays (Hi-C), overexpression of a ParA variant with higher affinity for ParB displayed a slight decrease in chromosomal arm alignment compared to overexpressing the wildtype *parA* (48). Changes in the compaction/organization level of the chromosome around *ori* could impact DnaA’s ability to initiate chromosome replication, which would be consistent with our findings. Tran et al. found no clear differences in the Hi-C maps between wildtype cells and cells overexpressing the wildtype version of *parA* (48). Aside from interfering with proteins that bind at or near *ori*, high levels of ParA could impact replication initiation by altering the regulation of cell length and/or cell division. In C. crescentus, altering cell wall biosynthesis (using cephalexin) or inhibition of cytokinesis (by FtsZ depletion) results in cells with *ori* supernumerary (49), a similar phenotype to the cells we examined with high levels of ParA. Further analyses are required to fully deduce the exact mechanism(s) involved in ParA’s impact on replication initiation in C. crescentus.

ParA’s activity depends on its nucleotide-dependent cycling between the monomeric and the DNA-bound dimeric states. In B. subtilis, Soj (ParA) can repress and activate replication initiation depending on its oligomeric state (monomeric versus dimeric) (15, 17, 45). The corresponding variant of G16V in Soj has been shown to directly inhibit DnaA’s activity (17). In C. crescentus, we show that ParA^G16V^ does not impact replication initiation positively or negatively. These differences in regulation are likely due to various differences in strategies that these bacteria use for ParA (Soj). For instance, C. crescentus and B. subtilis maintain their ParA (Soj) at different oligomeric states. In B. subtilis, Soj is found primarily in its monomeric state (45, 50), whereas ParA in C. crescentus is found primarily in the dimeric state (21). In summuary, our results reveal that, like B. subtilis, C. crescentus links the partitioning protein ParA to the onset of chromosome replication, albeit the specific mechanism (direct versus indirect) vary between these 2 diverse bacterial species.

Our work revealed that the deregulation of ParA levels in the cell results in the deregulation of the onset of chromosome replication in C. crescentus. Altering the levels of ParA have been shown to impact chromosome replication in Bacillus and V. cholera (15–17). Beyond chromosome replication, altering the levels of ParA can also impact other important functions like motility in Pseudomonas aeruginosa (51), and symbiosis in the nitrogen-fixing bacterium Azorhizobium caulinodans (52). These results would suggest that the levels of ParA must be tightly regulated. For the ParABS system that works on segregation of low copy plasmids, ParA or ParB, depending on the plasmid, have been shown to regulate the transcription of *parAB* (53–56). Less is known about the regulation of the ParAB system involved in chromosome segregation (57). In C. crescentus, the transcriptional levels and protein levels of ParA do not change over the cell cycle (58). The only reported system where the transcription of *parAB* are cell cycle regulated are in Streptomyces coelicolor during sporulation (59–61). It remains unclear whether outside Streptomyces, the cellular levels of ParA are cell cycle regulated.

Our work adds C. crescentus, with its dimorphic lifestyle, to the number of bacterial species that use the partitioning system to fine tune complex developmental processes. For instance, in B. subtilis, the par system is essential during sporulation where Soj (ParA) can directly interact with DnaA and inhibit the initiation of chromosome replication (15, 17). In bacteria with multipartite genomes like V. cholera, the par system has been shown to coordinate the segregation of each chromosome with the onset of chromosome replication (62, 63). In the stalk budding bacterium Hyphomonas neptunium, the par system is essential for regulating chromosome segregation in their unusual life cycle where replication and segregation of the chromosome are uncoupled (64). Although the exact mechanisms may vary among bacterial species, the strategy of using the partitioning system to coordinate other cell cycle events involved in maintaining the integrity of the chromosome seems to be conserved in evolutionarily diverse species.

## MATERIALS AND METHODS

### Strains and plasmids.

All the strains, plasmids, and primers used in this study are listed in Table S1, 2, and 3. Plasmids were constructed by cloning PCR amplified DNA fragments into pXCHYC-2, pXCHYC-5, pVCHYC-6, pVCHYC-2 (35), and pNPTS138 vectors. C. crescentus wild-type CB15N (NA1000) genomic DNA was used as the PCR DNA substrate. For bacterial two-hybrid assay, C. crescentus *dnaA* and *parA* genes were cloned into pKNT25 and pUT18 BACTH vectors containing fragments of adenylate cyclase (Euromedex BATCH System). Plasmids were transformed into E. coli DH5α cells and grown in Luria-Bertani medium (Fisher Bioreagents) at 37°C with orbital shaking at 200 rpm. All plasmid constructs were verified by sequencing.

### Growth conditions.

C. crescentus strains inoculated from freezer stocks were grown in minimal media (M2G) or rich media (PYE) (65) at 30°C and 180 rpm. Liquid media was supplemented with 5 μg/mL Kanamycin (Kanamycin sulfate in water, IBI Scientific), 0.2 μg/mL Chloramphenicol (in 100% ethanol), or 0.2 μg/mL Tetracycline (in 50% ethanol). PYE plates contained 25 μg/mL Kanamycin or 1 μg/mL Chloramphenicol. Unless otherwise stated, C. crescentus cells were synchronized using the mini-synchrony protocol to isolate swarmer cells (66). Briefly, cells grown in M2G (15 mL) to exponential phase (OD_600_ ~0.3) were pelleted by spinning at 6000 rpm for 10 min at 4°C. The cell pellet was resuspended in 800 μL of 1 × M2 salts, and then mixed with Percoll (900 mL, Sigma-Aldrich). The mixed solution, in a 2 mL microfuge tube, was centrifuged at 11,000 rpm for 20 min at 4°C to separate the swarmer cells via density gradient separation. The bottom layer containing swarmer cells was carefully extracted and added to a new tube, and washed twice with 1 × M2 salts, centrifuging at 8000 rpm for 3 min at 4°C. Isolated swarmer cells were resuspended in M2G medium (2 mL, OD600 ~0.1). Culture was supplemented with 0.1% xylose at *t* = 0 h, and incubated at 30°C in a roller-shaker. When pre-induction of ParA/HdaA was required, the inducer 0.1% xylose/250 mM van (or amounts as noted, Sigma-Aldrich) was added 1 h prior to synchrony. PYE media was used during C. crescentus strain constructions.

### Growth analyses.

Strains were inoculated from freezer stocks supplemented with Kan, and incubated overnight at 30°C. Saturated cultures grown in M2G were diluted in fresh media (200 mL) to OD_600_ ~0.01 in 96-well plates. Absorbance at OD_600_ was measured every hour in a Biotek EPOCH-2 microplate reader at 30°C with orbital shaking at 180 rpm.

### Microscopy.

Cells (~2 μL) were spotted on agarose pads (1% agarose in M2G). Agarose pads were supplemented with 0.1% xylose during time lapse imaging. Phase contrast fluorescence micrographs were obtained using the Zeiss Axio Observer 2.1 inverted microscope with AxioCam 506 mono camera (objective: Plan-Apochromat 100×/1.40 Oil Ph3 M27 [WD = 0.17 mm]), and Zen lite software. Number of CFP-ParB foci per cell was manually counted (Cell Counter Plugin), and cell length was analyzed using ImageJ/FIJI with MicrobeJ software (67). To visualize chromosomal DNA in live cells, cells were stained with 4,6-diamidino-2-phenylindole (DAPI) (Thermo Scientific) using a DNA staining protocol (68). Briefly, 1 mL of cells (OD_600_ ~0.4−0.6) were centrifuged at 7000 × *g* for 5 min, then resuspended in 1 mL of phospjate-buffered saline (PBS) (137 mM NaCl, 2.7 mM KCl, 8 mM Na_2_HPO_4_, 2 mM KH_2_PO_4_) containing 1 μL of DAPI stock solution (1 mg/1 mL), mixed by pipetting, then followed by a 5-min incubation in the dark. Cells were pelleted by centrifugation at 7000 × *g* for 5 min and resuspended in 1 mL of PBS. The washing step was repeated twice, followed by resuspension in 50 μL of M2G. Immediately after, ~2 μL of cells were applied to an agarose pad and imaged using epifluorescence microscopy.

### Immunoblotting.

C. crescentus swarmer cells were isolated using the mini-synchrony protocol, and the inducer was added (as specified in the figure legends). OD_600_ of incubated cultures were normalized to ~0.2, and the cell pellets resuspended in Cracking buffer (40 μL) were boiled at 80°C for 10 min and stored at –20°C for Western blots. Proteins were separated on SDS-PAGE and transferred into the nitrocellulose membrane using iBlot2 (Invitrogen Dry Blotting System). The membrane was blocked with 1 × TBS (10 mM Tris-Cl, pH 8.0, 150 mM NaCl) with 5% nonfat milk and 0.1% Tween 20 (T) for 1 h. The blot was probed with ~1:10,000 diluted primary antibody (anti-Flag for ParA-M2, F7425 Sigma-Aldrich, and anti-DnaA for DnaA) overnight at 4°C. The membrane was washed three times with 1 × TBST and incubated for 1 h with 1:10,000 diluted secondary antibody (Anti-Rabbit IgG peroxidase, Sigma-Aldrich) at room temperature. Excess secondary antibody was removed by washing the membrane with 1 X TBST three times. The membrane was developed with SuperSignal West Pico PLUS Chemiluminescent Substrate (Thermo Scientific), and imaged with ChemiDoc-MP imaging system (Bio-Rad Laboratories).

### Flow cytometry analysis.

Swarmer cells were isolated via mini-synchrony (66) and resuspended into M2G. At respective time points, aliquots were treated with a flow cytometry protocol previously described (69) to analyze chromosome content. In summary, cell cultures were incubated with Rifampicin (16 μg/mL in 100% methanol) to block the re-initiation of DNA replication. The samples were incubated for 3 h at 30°C. Afterward, they were fixed with 70% ethanol, and stored at 4°C for up to 24 h. The fixed cells were then spun down at 4000 × *g* and resuspended gently in TMS buffer (10 mM Tris-HCl pH 7.2, 1.5 mM MgCl_2_, 150 mM NaCl), and stained with 10 μM Vybrant DyeCycle Orange (Invitrogen). The fixed samples were analyzed on a BD LSR II Flow cytometer. Flow cytometry data were analyzed using BD FACSDiva software (BD Biosciences).

### Bacterial two-hybrid assay.

DHM1 cells from freezer stock were streaked on a LB plate supplemented with Nalidixic acid (30 mg/mL), and incubated over night at 30°C. Colonies were resuspended in 1 mL of sterile water and washed 3 times with water prior to the co-transformation of plasmids using the electroporator. + control, vector control, and the DnaA/ParA test strains were created by co-transforming pKT25-zip/pUT18C-zip, pKT25/pUT18C, and pUT18-ParA/pKNT25-DnaA plasmid combinations. Cells were plated on LB agar supplemented with IPTG (0.5 mM), X-gal (40 mg/mL), Nalidixic acid (30 mg/mL), Kanamycin (50 mg/mL), and Ampicillin (100 mg/mL), and incubated over night at 30°C. A single colony of each strain was inoculated in 2 mL of LB media supplemented with Kanamycin (50 mg/mL) and Ampicillin (100 mg/mL), and incubated at 30°C. Cultures at early log phases were diluted to 0.1 OD_600_, and spotted using 5 mL on LB agar supplemented with IPTG, X-gal, Nalidixic acid, Kanamycin, and Ampicillin, and incubated overnight at 30°C.
