# Supplementary material for: ParA’s Impact beyond Chromosome Segregation in Caulobacter crescentus
Source: J Bacteriol. 2023 Jan 24;205(2):e00296-22. doi: 10.1128/jb.00296-22 (PMC9945499; doi:10.1128/jb.00296-22)
Supplement: Supplemental file 1 — Fig. S1 to S5 and Tables S1 to S3. Download jb.00296-22-s0001.pdf, PDF file, 0.6 MB [file jb.00296-22-s0001.pdf]

Supplementary Data

ParA's impact beyond chromosome segregation in *Caulobacter crescentus*

Inoka P. Menikpurage[1,§], Stephanie G. Puentes-Rodriguez[1,§], Rawan A. Elaksher[2], and Paola E. Mera[1,*]

[1]Department of Microbiology, University of Illinois at Urbana-Champaign, Urbana, IL, USA
[2]Department of Chemistry and Biochemistry, New Mexico State University, Las Cruces, NM, USA
[§]These authors contributed equally: I. P. Menikpurage and S. G. Puentes-Rodriguez.
*e-mail: pmera@illinois.edu

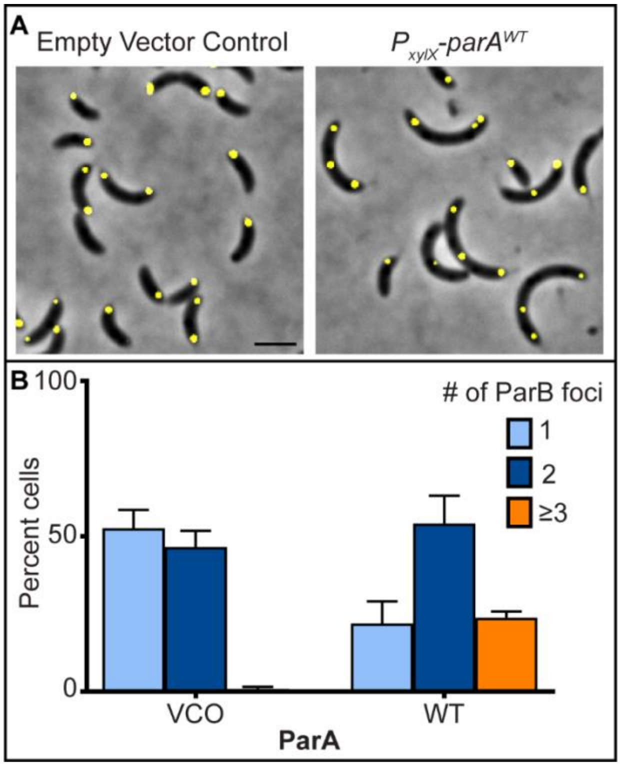

Figure S1: Supernumerary of *ori* regions in *cc0006::(tetO)n (gent^R), P$_{van}$::tetR-eyfp C. crescentus* cells overexpressing ParA^{WT}. (A) *C. crescentus cc0006::(tetO)n (gent^R), P$_{van}$::tetR-eyfp* swarmer cells having empty vector or *xylX::parA^{WT}* in M2G (2 ml) supplemented with 0.1% xylose were incubated at 30°C in a roller-shaker. Phase-contrast fluorescence micrographs of cells expressing ParA^{WT} obtained at 4 h show more than 2 TetR-eYFP yellow foci corresponding to multiple *oris* compared to empty vector control with 2 TetR-eYFP foci. Micrograph scale bar – 2 µm. **(B)** Bar graphs of the percent number of tetR-eyfp foci (oris) per cell. Data shown are representative of three independent experiments with error bars of mean ± standard deviation (SD).

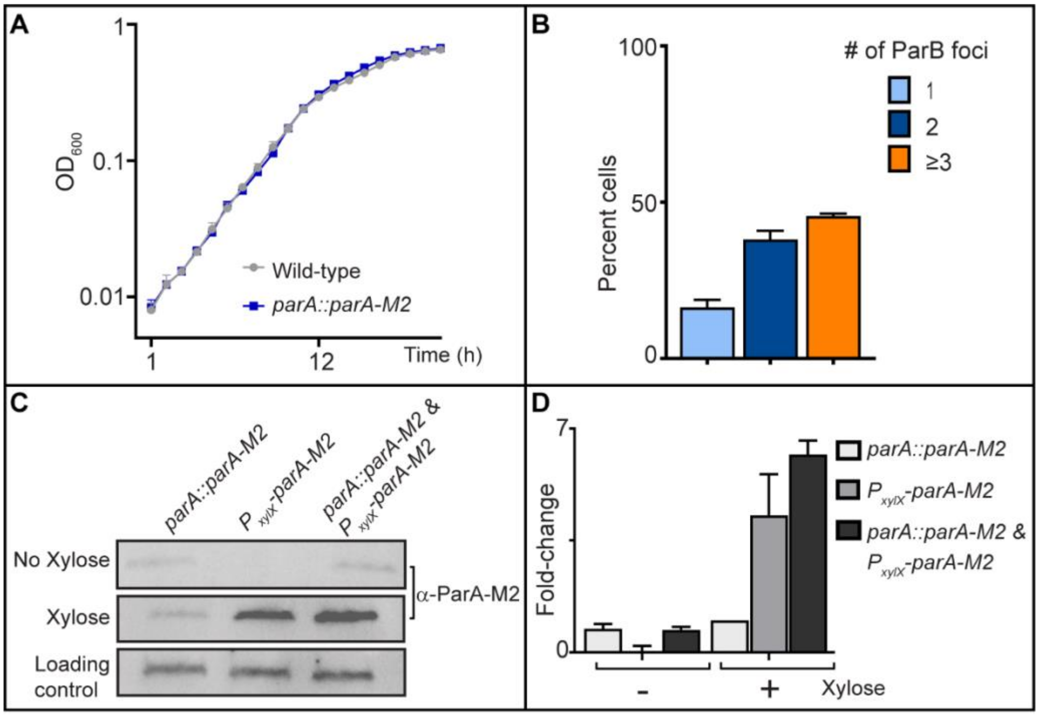

Figure S2: Characterization of ParA-M2 and overexpression levels. (A) Growth curves of *C. crescentus* CB15N (wild-type) and *parA::parA-M2* cells in M2G media. **(B)** Quantification plot of CB15N, *xylX::parA-M2* cells expressing ParA^{WT}-M2 in the presence of xylose (0.1 %) for 3 h. **(C)** Western blots of ParA-M2 levels expressed under native promoter (no xylose) and in the presence of xylose after 3 h. **(D)** Bar graph of quantfication data of western blots **(C)**. **A** and **C** - Data represent three independent experiments. **B** and **C** data are from three independent experiments with error bars of mean ± standard deviation (SD).

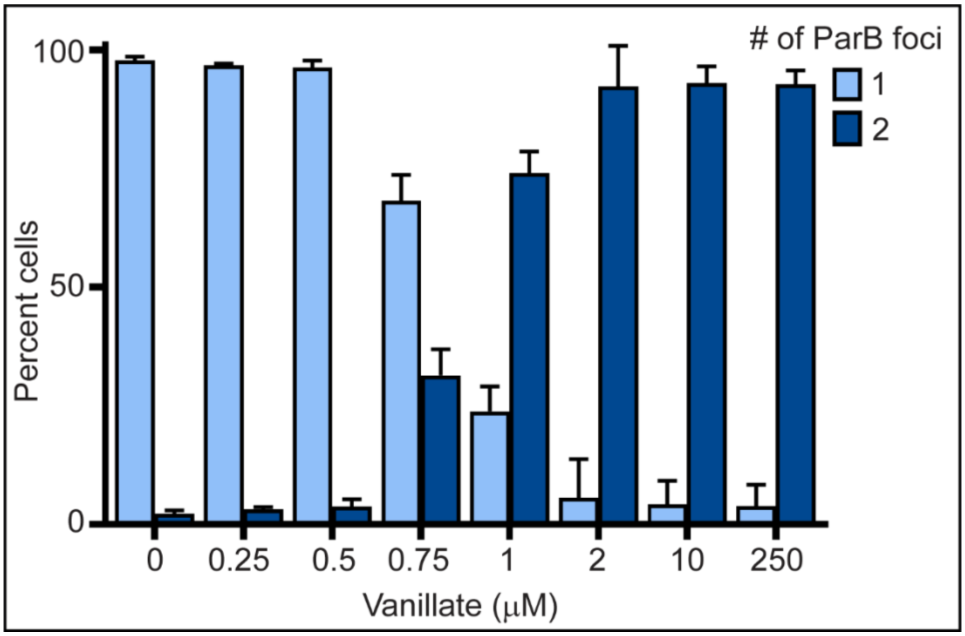

Figure S3: Quantification of frequencies of replication initiation in cells with titrated *dnaA* induction. Bar graph of percent of replication initiation of *C. crescentus* CB15N, *parB*::*cfp-parB,* Δ*vanA, dnaA*::Ω, *vanA*::*dnaA* cells under increasing amounts of vanillate (0 - 250 μM) to induce DnaA. Swamer cells in M2G (2 ml) were incubated at 30°C in a roller-shaker for 3 h to deplete DnaA and added increasing amounts of vanillate (0, 0.25, 0.5, 0.75, 1, 2, 10 and 250 μM) to induce DnaA expression. Micrographs were obtained after 1 h incubation and the number of CFP-ParB per cell was quantified. Data are from three independent experiments with error bars of mean ± standard deviation (SD).

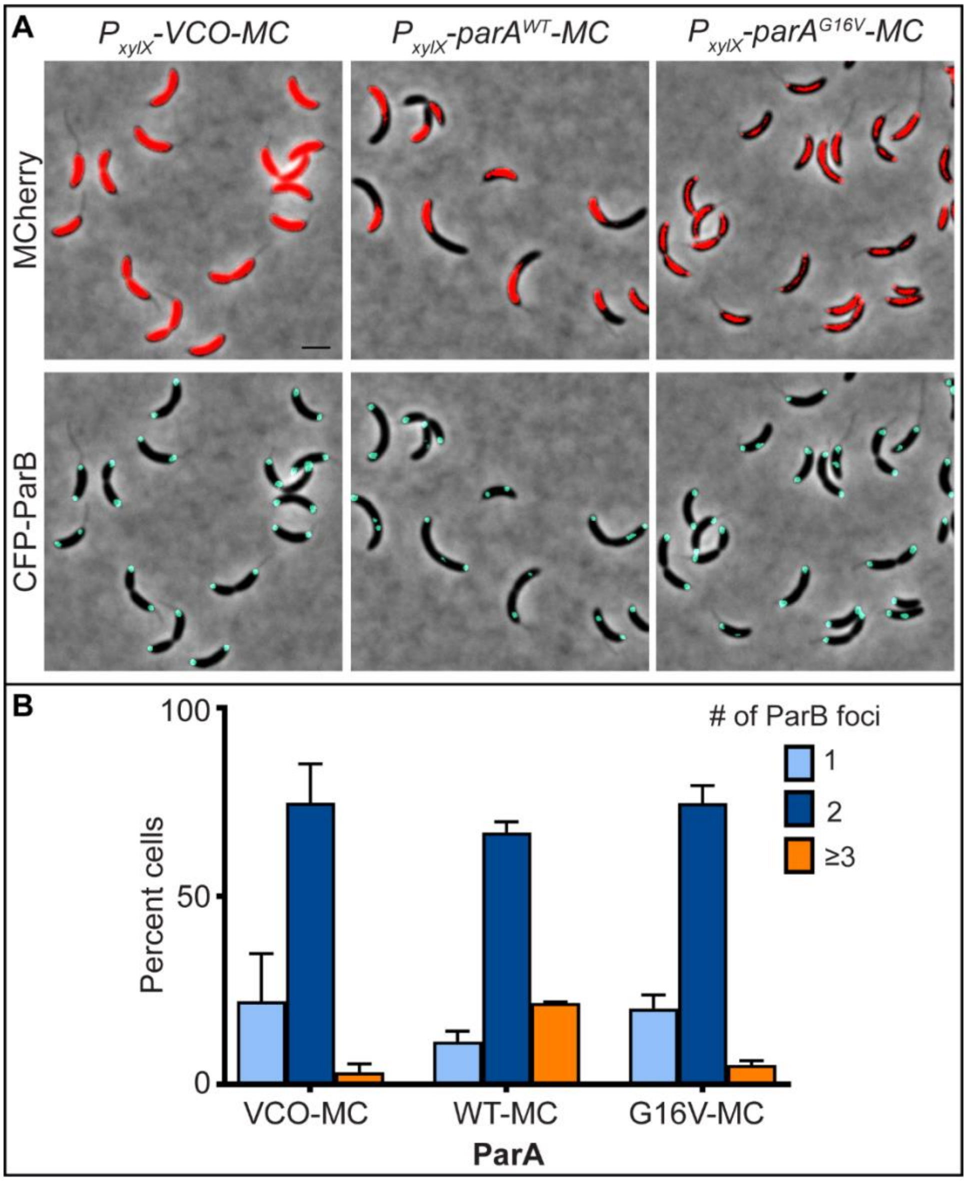

Figure S4: *C. crescentus* cells express and accumulate ParA^{G16V}-MC in the cell. (A) Mix population of *C. crescentus* CB15N, *parB::cfp-parB* cells having empty vector-mCherry (MC), *xylX::parA^{WT}*-MC, or *xylX::parA^{G16V}*-MC grown in M2G (2 ml) supplemented with 0.1% xylose were incubated at 30°C in a roller-shaker. Phase-contrast fluorescence micrographs of cells were obtained at 3 h. MCherry tag proteins expressed in vector control (top-left) are dispersed throughout the cell, ParA^{WT}-MC forms the gradient (top-center), and ParA^{G16V}-MC (right) is dispersed with foci localized at the poles. Micrograph scale bar – 2 μm. All samples were imaged and adjusted to the same fluorescence intensities. **(B)** Bar graphs of the percent number of ParB foci (*oris*) per cell. Data shown are representative of three independent experiments with error bars of mean ± standard deviation (SD).

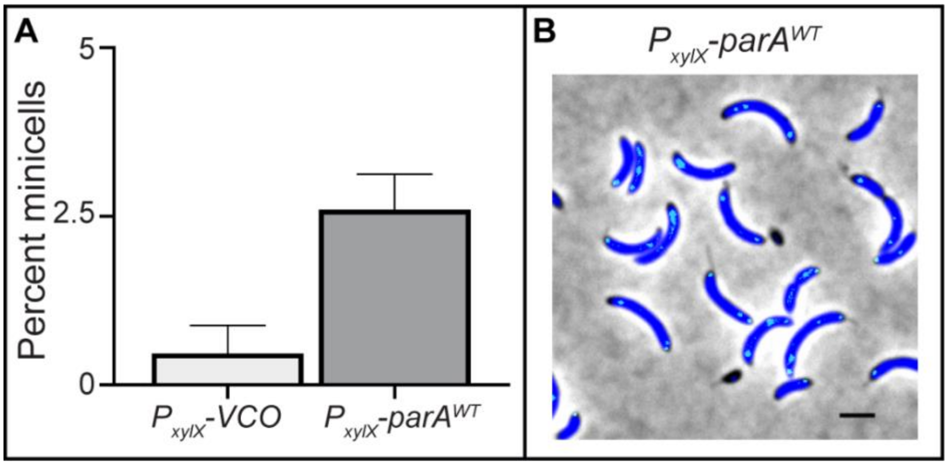

Figure S5: Minicells derived from ParA^{WT} overexpression do not contain DNA. (A) Bar graph of percent mini-cells in cultures of CB15N *parB∷cfp-parB* cells of *xylX::VCO* (empty vector control) and *xylX::parA*^{WT} grown in M2G + xylose (0.1 %) after 3h. (B) Micrograph of *C. crescentus* CB15N, *parB∷cfp-parB, xylX::parA*^{WT} cells expressing ParA^{WT} under the xylose promoter for 3 hr, then stained with DAPI (blue) to visualize the chromosome. Micrograph scale bar – 2 μm.

Table 1: List of strains used in this study.

Name	Relevant genotype or description	Reference
***C. crescentus* strains**		
	CB15N (NA1000)	[1]
PM109	CB15N, *parB*::*cfp-parB,* Δ*vanA*, *dnaA*::Ω (strp^{R}/spec^{R}), *vanA*::*dnaA*	[2]
PM258	CB15N, *parA::parA-M2*	This study
	CB15N, *parB*::*cfp-parB*	[2]
PM359	CB15N, *parB*::*cfp-parB xylX::;mCherry*	This study
PM502	CB15N, *parB*::*cfp-parB, xylX::parA-mCherry* (kan^{R})	This study
PM541	CB15N, *parB*::*cfp-parB, xylX::parA*^{WT} (kan^{R})*, parB::cfp-parB*	This study
PM542	CB15N, *parB*::*cfp-parB,* Δ*vanA*, *dnaA*::Ω (strp^{R}/spec^{R}), *vanA*::*dnaA, xylX::parA*^{WT} (kan^{R})	This study
PM550	CB15N, *parB*::*cfp-parB, xylX::parA-(D44A)* (kan^{R})	This study
PM566	CB15N, *parB*::*cfp-parB, xylX::empty-vector* (kan^{R})	This study
PM567	CB15N, *parB*::*cfp-parB, xylX:: parA-(WT)-M2* (kan^{R})	This study
PM607	CB15N, *parB*::*cfp-parB,* Δ*vanA*, *vanA*::hda*A* (chlor^{R})*, xylX::parA*^{WT} (kan^{R})	This study
PM656	CB15N, *parB*::*cfp-parB, xylX::parA-(R195A)* (kan^{R})	This study
KJ300	CB15N *cc0006::(tetO)*$_{n}$ (gent^{R})+P*van::tetR-eyfp* (spec^{R}) (gent^{R} , spec^{R})	[3]
PM714	CB15N, *xylX::parA-M2* (kan^{R})	This study
PM718	CB15N *parA::parA-M2, xylX::parA-M2* (kan^{R})	This study
PM741	CB15N *cc0006::(tetO)*$_{n}$ (gent^{R})+P*van::tetR-eyfp* (spec^{R}) (gent^{R} , spec^{R}), *xylX::parA-(WT)* (kan^{R})	This study
PM750	CB15N, *parB*::*cfp-parB, ΔparA*, *xylX::parA-M2* (chlor^{R})	This study
PM767	CB15N, *parB::cfp-parB, xylX::parA-(G16V)* (kan^{R})*,*	This study
PM771	CB15N, *parB*::*cfp-parB,* Δ*vanA*, *dnaA*::Ω (strp^{R}/spec^{R}), *vanA*::*dnaA, xylX::empty-vector* (kan^{R})	This study
PM818	CB15N, *parB*::*cfp-parB, xylX::parA-(G16V)-mCherry* (kan^{R})	This study
PM821	CB15N, *parB::cfp-parB, xylX::parA-(K20A)* (kan^{R})	This study
PM864	CB15N *popZ::popZ-YFP, xylX::empty-vector* (kan^{R})	This study
PM865	CB15N *popZ::popZ-YFP, xylX::parA(WT)* (kan^{R})	This study
***E. coli* strains**		
DHM1	Reporter strain for BACTH assay	Euromedex, Cat #EUK001

Table 2: List of plasmids used in this study.

Plasmid name	Description	Reference
pNPTS138	Nonreplicating vector for allelic replacement (kan^R or chlor^R) *oriT sacB*	Alley M. R. K., unpublished
pXCHYC-2	Integrating constructs encoding C-terminal mCherry fusions under the control of native P_{xylX} (kan^R)	[4]
pXCHYC-5	Integrating constructs encoding C-terminal mCherry fusions under the control of native P_{xylX} (tet^R)	[4]
pVCHYC-2	Integrating constructs encoding C-terminal mCherry fusions under the control of native P_{vanA} (kan^R)	[4]
pVCHYC-6	Integrating constructs encoding C-terminal mCherry fusions under the control of native P_{vanA} (chlor^R)	[4]
pDNA91	*parA-mCherry* cloned into pXCHYC-2 (kan^R)	This study
pDNA245	*parA* cloned into pXCHYC-2 (kan^R)	This study
pDNA255	pNPTS138 derivative to replace *parA* by *parA-M2* under the native P_{parA} (kan^R)	This study
pDNA257	*parA-(D44A)* cloned into pXCHYC-2 (kan^R)	This study
pDNA264	*mCherry* tag excised from pXCHYC-2 (kan^R)	This study
pDNA274	*hdaA* cloned into pVCHYC-6 (chlor^R)	This study
pDNA310	*parA-(R195A)* cloned into pXCHYC-2 (kan^R)	This study
pDNA315	*parA-M2* cloned into pXCHYC-2 (kan^R)	This study
pDNA321	pNPTS138 derivative to delete native *parA* while keeping the downstream *cfp-parB* (kan^R)	This study
pDNA323	*parA-M2* cloned into pXCHYC-6 (chlor^R)	This study
pDNA329	*parA-(G16V)* cloned into pXCHYC-2 (kan^R)	This study
pDNA342	*parA-(G16V)-mCherry* cloned into pXCHYC-2 (kan^R)	This study
pDNA350	*parA-(K20A)* cloned into pXCHYC-2 (kan^R)	This study
pDNA362	*dnaA* cloned into pKNT25 BACTH vector (kan^R)	This study
pDNA363	*parA* cloned into pUT18 BACTH vector (Amp^R)	This study

Table 3. List of oligonucleotides used in this study.

Plasmid name	Oligonucleotide sequence (5'→3')	Use
pDNA91	Fwd - AAAGAGCTCTGGCGGCCTTGGCCTG<br>Rev - AAACATATGTCCGCTAATCCTCTCCG	*parA-mCherry* cloned into pXCHYC-2
pDNA245	Fwd - AAAGAGCTCTGGCGGCCTTGGCCTG<br>Rev - AAAGCTAGCTTAGGCGGCCTTGGCCTG	*parA* cloned into pXCHYC-2
pDNA255	UP 800 bp fwd – CGCCAAGCTTCTCTGCAGGATATCTGGATCATGTCCGCTAATCCTCTCCG<br>UP 800 bp rev - TTACTTGTCATCGTCATCCTTGTAGTCGGCGGCCTTGGCCTGGCGATC<br>DWN 500 bp fwd - CGCCGACTACAAGGATGACGATGACAAGTAAGTCCAAAGAACAAGAACCGTAGC<br>DWN 500 bp rev - CGCGTCACGGCCGAAGCTAGCGAATTCGTGCAGCACCTTGTAGGAGAGCGC	pNPTS138 derivative to replace *parA* by *parA-M2* under the native P_{parA} via Gibson assembly
pDNA257	Fwd - CTGATCGACGCCGCCCCGCAGGGCAAC<br>Rev - GTTGCCCTGCGGGGCGGCGTCGATCAG	*parA-(D44A)* cloned into pXCHYC-2 via site directed mutagenesis using pDNA245
pDNA264	Fwd - TCGAGTTTTGGGGAGACGACCATATGTGCAGCCCGGGGGATCC<br>Rev - GTGCTGCAAGGCGATTAAG	*mCherry* tag excised from pXCHYC-2 via Gibson assembly
pDNA274	Fwd - AAACATATGTTGTCCACCCAGTTCAA<br>Rev - AAAGCTAGCCTACCCCTCATCCCCCTCG	*hdaA* cloned into pVCHYC-6
pDNA310	Fwd - CCATGTACGACCGCGCCAACAGCTTGT<br>Rev - CAAGCTGTTGGCGCGGTCGTACATGGTC	*parA-(R195A)* cloned into pXCHYC-2 via site-directed mutagenesis
pDNA315	Fwd - AAACATATGTCCGCTAATCCTCTCCG<br>Rev - AAAGCTAGCTTACTTGTCATCGTCATC	*parA-M2* cloned into pXCHYC-2
pDNA323	Fwd - AAAGAGCTCTGGCGGCCTTGGCCTG<br>Rev- -AAAGCTAGCTTACTTGTCATCGTCATC	*parA-M2* cloned into pXCHYC-6
pDNA329	Fwd - GCCAATCAAAAGGTTGGGGTGGGGAAG<br>Rev - CTTCCCCACCCCAACCTTTTGATTGGC	*parA-(G16V)* cloned into pXCHYC-2 via site-directed mutagenesis
pDNA362	Fwd - AAAAAAGGTACCATGACCATGAAGGGCGGGG<br>Rev - AAAGAATTCGCCCCGCAGCTTGCGCGTC	*dnaA* cloned into pKNT25 BACTH vector
pDNA363	Fwd - AAAGGTACCATGTCCGCTAATCCTC<br>Rev - AAAGAATTCGGCGGCCTTGGCCTGGCG	*parA* cloned into pUT18 BACTH vector

REFERENCES

1. Evinger, M. and N. Agabian, *Caulobacter crescentus nucleoid: Analysis of sedimentation behavior and protein composition during the cell cycle.* Proceedings of the National Academy of Sciences, 1979. **76**: p. 175-178.
2. Mera, P.E., V.S. Kalogeraki, and L. Shapiro, *Replication initiator DnaA binds at the Caulobacter centromere and enables chromosome segregation.* Proceedings of the National Academy of Sciences, 2014. **111**(45): p. 16100-16105.
3. Jonas, K., Y.E. Chen, and M.T. Laub, *Modularity of the Bacterial Cell Cycle Enables Independent Spatial and Temporal Control of DNA Replication.* Current Biology, 2011. **21**(13): p. 1092–1101.
4. Thanbichler, M., A.A. Iniesta, and L. Shapiro, *A comprehensive set of plasmids for vanillate- and xylose-inducible gene expression in Caulobacter crescentus.* Nucleic Acids Research, 2007. **35**(20): p. e137-e137.
5. Toro, E., et al., *Caulobacter requires a dedicated mechanism to initiate chromosome segregation.* Proceedings of the National Academy of Sciences, 2008. **105**(40): p. 15435-15440.
